# Characteristics of β-lactamases and their genes (*blaA *and *blaB*) in *Yersinia intermedia *and *Y. frederiksenii*

**DOI:** 10.1186/1471-2180-7-25

**Published:** 2007-04-03

**Authors:** Shilpi Mittal, Sarita Mallik, Sachin Sharma, Jugsharan S Virdi

**Affiliations:** 1 Microbial Pathogenicity Laboratory, Department of Microbiology, University of Delhi South Campus, Benito Juarez Road, New Delhi 110 021, India

## Abstract

**Background:**

The presence of β-lactamases in *Y. enterocolitica *has been reported to vary with serovars, biovars and geographical origin of the isolates. An understanding of the β-lactamases in other related species is important for an overall perception of antibiotic resistance in yersiniae. The objective of this work was to study the characteristics of β-lactamases and their genes in strains of *Y. intermedia *and *Y. frederiksenii*, isolated from clinical and non-clinical sources in India.

**Results:**

The enzymes, Bla-A (a constitutive class A penicillinase) and Bla-B (an inducible class C cephalosporinase) were found to be present in all the clinical and non-clinical strains of *Y. intermedia *and *Y. frederiksenii *by double disc diffusion method. The results showed differential expression of Bla-A as indicated by presence/absence of synergy whereas expression of Bla-B was quite consistent. The presence of these enzymes was also reflected in the high minimum inhibitory concentrations, MIC_50 _(126–1024 mg/L) and MIC_90 _(256–1024 mg/L) of β-lactam antibiotics against these species. Restriction fragment length polymorphism (RFLP) revealed heterogeneity in both *blaA *and *blaB *genes of *Y. intermedia *and *Y. frederiksenii*. The *blaA *gene of *Y. intermedia *shared significant sequence identity (87–96%) with *blaA *of *Y. enterocolitica *biovars 1A, 1B and 4. The sequence identity of *blaA *of *Y. frederiksenii *with these biovars was 77–79%. The sequence identity of *blaB *gene of *Y. intermedia *and *Y. frederiksenii *was more (85%) with that of *Y. enterocolitica *biovars 1A, 1B and 2 compared to other species *viz*., *Y. bercovieri*, *Y. aldovae *and *Y. ruckeri*. Isoelectric focusing data further revealed that both *Y. intermedia *and *Y. frederiksenii *produced Bla-A (pI 8.7) and "Bla-B like" (pI 5.5–7.1) enzymes.

**Conclusion:**

Both *Y. intermedia *and *Y. frederiksenii *showed presence of *blaA *and *blaB *genes and unequivocal expression of the two β-lactamases. Limited heterogeneity was detected in *blaA *and *blaB *genes as judged by PCR-RFLP. Phylogenetic relationships showed that the two species shared a high degree of identity in their *bla *genes. This is the first study reporting characteristics of β-lactamases and their genes in strains of *Y. intermedia *and *Y. frederiksenii *isolated from Asian region.

## Background

The genus *Yersinia*, a highly heterogenous organism is currently represented by eleven known species [1]. *Y. pestis, Y. pseudotuberculosis *and *Y. enterocolitica *are very well documented human pathogens. *Y. pestis *is the etiologic agent of plague (black death) while the other two are known to cause a variety of gastrointestinal syndromes [2]. The remaining eight species namely *Y. intermedia*, *Y. frederiksenii*, *Y. kristensenii*, *Y. bercovieri*, *Y. mollaretii*, *Y. aldovae*, *Y. rohdei *and *Y. ruckeri *have generally been termed as "*Y. enterocolitica*-like" species though each is a distinct species. Although most commonly isolated from sources like fresh water and food, these have only been infrequently recovered from human clinical specimens [1]. But, some of these, especially *Y. intermedia *and *Y. frederiksenii *may constitute as much as 18–32% of all *Yersinia *isolated from stools of diarrheic patients [3]. However, not much information is available about these, and Sulakvelidze termed them as the ignored species [1]. Nevertheless, *Y. intermedia *[4], *Y. frederiksenii *[5], *Y. bercovieri *[6] and *Y. kristensenii *[7] have been strongly implicated in cases of diarrhoea. It is therefore important to know more about these species to understand their public health significance.

The production of β-lactamases is an important mechanism of resistance to β-lactam antibiotics. In *Y. enterocolitica*, the distribution and production of β-lactamases namely Bla-A (a constitutive class A enzyme) and Bla-B (an inducible class C cephalosporinase or AmpC) has been studied widely and reported to vary with biovars and geographical origin of the strains [8-11]. However, not much is known about the β-lactamases of other important species like *Y. intermedia*, *Y. frederiksenii *and *Y. kristensenii*. Recently, *Y. intermedia*, *Y. frederiksenii*, and some strains of *Y. kristensenii *and *Y. rohdei *were reported to produce two β-lactamases [12,13]. On the other hand, *Y. bercovieri *and *Y. mollaretii *have been reported to produce only AmpC β-lactamase, while *Y. aldovae *and *Y. ruckeri *showed either low levels of AmpC or no expression of β-lactamases at all [14]. This information has been inferred from MIC data of β-lactam antibiotics. Schiefer et al [15] recently characterized β-lactamases of *Y. frederiksenii, Y. bercovieri, Y. aldovae *and *Y. ruckeri *and reported that except for *Y. frederiksenii*, all expressed only an AmpC β-lactamase. Mammeri et al [16] cloned and sequenced the complete *ampC *gene of *Y. ruckeri *and found that it shared low level of identity with the known chromosomal and plasmid AmpC enzymes of closely related members, *viz*., *Enterobacter cloacae, E. aerogenes *and *Citrobacter freundii*. Much of these data however pertains to strains isolated in Europe. It would be worthwhile to study the β-lactamases and *bla *genes of strains isolated from other parts of the world to understand the global drug resistance of yersiniae. No data is available on β-lactamases of "*Y. enterocolitica*-like" species isolated from Asian region. Therefore, the objective of this work was to detect and characterize the β-lactamases and *bla *genes of *Y. intermedia *and *Y. frederiksenii *isolated from clinical and non-clinical sources in India.

## Results and discussion

### Phenotypic detection of Bla-A and Bla-B enzymes

All the clinical and non-clinical isolates of *Y. intermedia *and *Y. frederiksenii *showed presence of Bla-A as indicated by the appearance of a small zone of inhibition of 2–8 mm radii (10–22 mm diameter) around ticarcillin 75 μg disc (Table 1). However, synergy, the appearance of a characteristic additional zone of inhibition between ticarcillin and the adjacent clavulanate disc, was detected in only 26% (9/34) of the isolates of *Y. intermedia *and one isolate of *Y. frederiksenii*. In an earlier study, similar observations regarding synergy were made for strains of *Y. enterocolitica *biovar 1A [8]. In the present study, Bla-B, the inducible cephalosporinase, was detected unequivocally based on the characteristic flattening of inhibition zone around cefotaxime disc adjacent to the imipenem disc in all the strains (Table 1). Tzelepi et al [17] also reported detection of a broad spectrum penicillinase (Bla-A) and an inducible cephalosporinase (Bla-B) in all the thirty aquatic isolates of *Y. intermedia*. Our study extends this information further to indicate that Bla-A was also present in *Y. intermedia *isolated from clinical and non-clinical (pig/wastewater) sources. In addition, the present study also showed unequivocal presence of Bla-A and Bla-B in strains of *Y. frederiksenii*, which was inferred earlier on the basis of MIC data only [12,13].

For Bla-A, a larger zone of inhibition (4–8 mm) around ticarcillin disc (75 μg) was observed for wastewater isolates of *Y. intermedia *compared to that of clinical (1.5–2.0 mm) isolates. The zone of inhibition (1.5–8 mm) around *Y. intermedia *was nevertheless larger compared to that around the strains of *Y. frederiksenii *(1.5–3.6 mm). Also, synergy *i.e*., appearance of an additional zone of inhibition around ticarcillin disc, was observed in the clinical isolates only. As these phenomena are highly dependent on the degree of the expression of enzyme, these observations suggested differential expression of Bla-A as reported earlier for strains of *Y. enterocolitica *biovar 1A [8].

### Minimum Inhibitory Concentration (MIC)

All the strains were resistant to penicillins and cephalosporins tested. When analyzed separately, no difference was observed in the antibiotic susceptibilities of *Y. intermedia *and *Y. frederiksenii*. Thus, the combined results are shown in Table 2. The MIC_50 _of amoxicillin was 512 mg/L for the clinical and 1024 mg/L for the non-clinical strains, whereas MIC_90 _was 1024 mg/L and 2048 mg/L, respectively. For co-amoxiclav, the MIC_50 _ranged from 128–256 mg/L and MIC_90 _was 256–512 mg/L for both clinical and non-clinical strains. The strains were equally resistant to cephalosporins. Amongst these, minimum resistance (MIC_50 _32–64 mg/L and MIC_90 _128 mg/L) was seen against cefotaxime for strains of *Y. intermedia *and *Y. frederiksenii*. For ceftazidime, the MIC_50 _and MIC_90 _were 512 mg/L and 1024 mg/L respectively, and for cefepime, the values ranged from 256–1024 mg/L. Contrary to these, relatively lower MICs of penicillins and cephalosporins have been reported for *Y. intermedia *and *Y. frederiksenii *isolated in other parts of the world [13,17,18]. This may be due to difference in the sources from which organisms were isolated or their geographical origin. Interestingly, Tzelepi et al [17] found that the strains of *Y. intermedia *isolated in Europe were sensitive to both cefotaxime and ceftazidime. The antibiotic resistance data of strains of *Y. intermedia *and *Y. frederiksenii *was in accordance with the preliminary reports from our laboratory earlier [19].

### PCR amplification and restriction digestion of *blaA *gene

The β-lactamase genes namely the *blaA *and *blaB *were detected by PCR amplification and found to be present in all strains of *Y. intermedia *and *Y. frederiksenii*.

Initially, an attempt was made to amplify *blaA *using published primers (A9-f and A10-r) of *Y. enterocolitica *biovar 1A [20]. However, though some of the strains of *Y. intermedia *yielded expected amplicon, none of the *Y. frederiksenii *strains gave any amplicon. This may be attributed to differences in the gene sequences of *blaA *of *Y. intermedia *and *Y. frederiksenii *compared to that of *Y. enterocolitica *biovar 1A. Thus, to amplify *blaA *of *Y. intermedia *and *Y. frederiksenii*, new internal primers (A7-f and A8-r) were designed. When these primers were used for amplification of *blaA*, the expected 450 bp amplicon was obtained for all the forty-nine strains of *Y. intermedia *and *Y. frederiksenii*.

**Figure 1:**
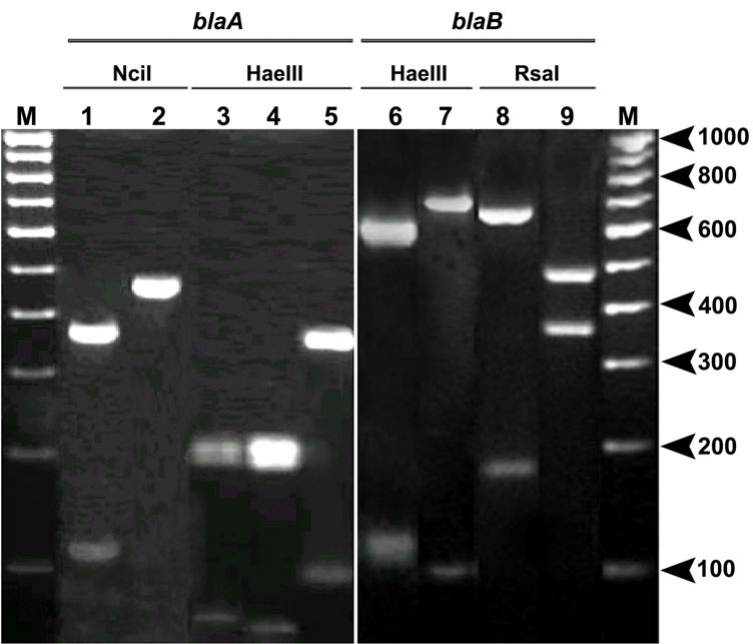
**Types of restriction profiles of *blaA *with NciI (Lanes 1–2) and HaeIII (Lanes 3–5), and *blaB *with HaeIII (Lanes 6–7) and RsaI (Lanes 8–9). **PCR amplification and restriction analysis were carried out for 49 strains of *Y. intermedia *and *Y. frederiksenii*. M, molecular weight DNA marker (100 bp DNA ladder)

**Figure 2:**
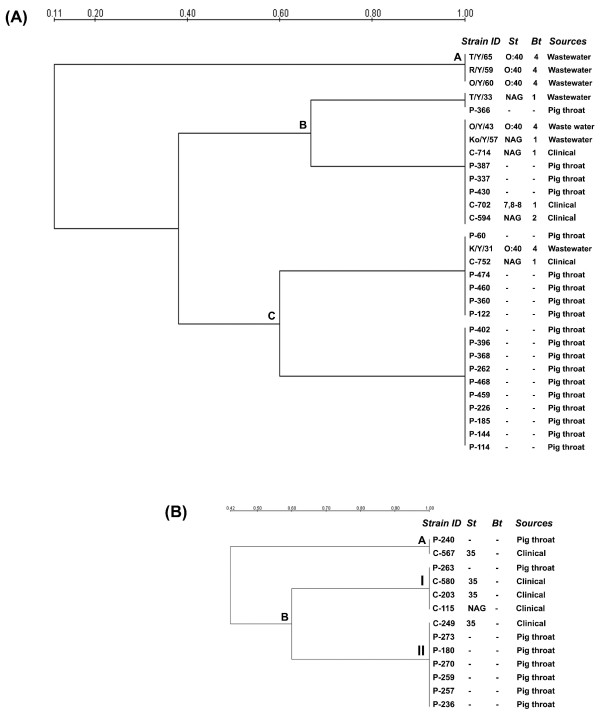
**Phylogenetic analysis of *blaA *gene of (A)*Y. intermedia *and (B) *Y. frederiksenii*. **NAG, non-agglutinable; -, Not determined; St, Serotype; Bt, Biotype

Restriction digestion of *blaA *with NciI revealed that a single site for NciI was present in *blaA *of only a few strains of *Y. intermedia *and *Y. frederiksenii *as either an uncut DNA or two fragments of 350 bp and 100 bp were obtained (Fig. 1). However, restriction with HaeIII gave three types of patterns having molecular weights 210, 190 and 60 bp (pattern I), 210, 190 and 55 bp (pattern II), and 350 and 100 bp (pattern III), for both *Y. intermedia *and *Y. frederiksenii*. The phylogenetic relationship and genetic heterogeneity in *blaA *was studied by constructing a concatenated dendrogram (Fig. 2) of the NciI and HaeIII restriction profiles. The strains of *Y. intermedia *grouped into three major clusters: A, B and C. Cluster A was formed by three wastewater isolates (T/Y/65, R/Y/59 and O/Y/60, all of serovar O:40). Cluster B was most heterogenous comprising clinical, wastewater and pig throat isolates while the cluster C was represented predominantly by pig throat isolates (Fig. 2a). The clusters B and C were divided further into two subclusters each. The *blaA*-RFLP grouped strains of *Y. frederiksenii *into two major clusters – A and B (Fig. 2b). Except for one, all the strains of clinical origin belonged to cluster B. As observed for *Y. intermedia*, the strains of *Y. frederiksenii *isolated from pig throat also clustered separately into subgroup BII (Fig. 2b). Earlier, work from our laboratory reported that some clinical and non-clinical strains of *Y. enterocolitica *biovar 1A formed separate clusters based on *blaA*-RFLP [20].

Several reports indicate that genetic background of antibiotic resistance genes influence antibiotic susceptibility profiles [21,22]. However, no such unequivocal relationship between *blaA-*RFLP type and antibiotic susceptibility was observed in this study. The present work however, clearly revealed heterogeneity in *blaA *gene of *Y. intermedia *and *Y. frederiksenii*, which may account for differential expression of Bla-A enzyme as seen in double disc diffusion method. Nevertheless, the heterogeneity was only limited as observed earlier for *blaA *gene of *Y. enterocolitica *biovar 1A strains [20]. In *Moraxella catarrhalis*, the two class A β-lactamase genes namely BRO-1 and BRO-2, have been reported to give different restriction profiles with BcgI [23,24].

### PCR amplification and restriction digestion of *blaB *gene

**Figure 3:**
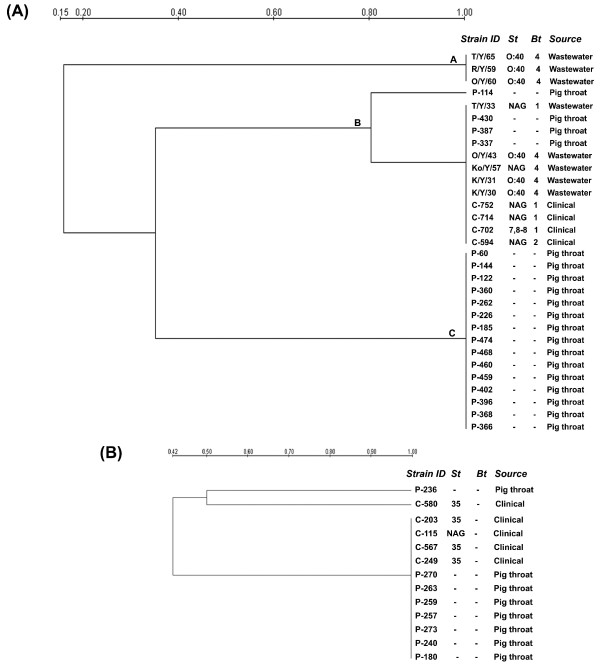
**Phylogenetic analysis of *blaB *gene of (A)*Y. intermedia *and (B) *Y. frederiksenii*. **NAG, non-agglutinable; -, Not determined; St, Serotype; Bt, Biotype

PCR amplification of *blaB *gene resulted in 850 bp product for all the forty-nine strains of *Y. intermedia *and *Y. frederiksenii*. The restriction digestion of *blaB *of *Y. intermedia *and *Y. frederiksenii *with HaeIII gave two types of patterns *i.e*., 700 bp and 100 bp, and 600 bp and 120 bp (Fig. 1). With RsaI, except for the three wastewater isolates of *Y. intermedia *(T/Y/65, R/Y/59 and O/Y/60), all strains of both the species showed identical pattern, with fragment sizes of 650 bp and 195 bp. The concatenated dendrogram of *blaB*-RFLP grouped the strains of *Y. intermedia *into three major clusters: A, B and C. As with *blaA*, except for the three wastewater isolates (T/Y/65, R/Y/59 and O/Y/60), all the clinical and majority of the wastewater isolates grouped together in cluster B, whereas group C consisted exclusively of pig throat isolates (84%) (Fig. 3a). Cluster analysis of restriction profiles of *Y. frederiksenii *revealed that except for two, all clinical and pig throat strains grouped together in one major cluster (Fig. 3b). In a previous study, when the same restriction enzymes (HaeIII and RsaI) were used, no heterogeneity was discerned in *blaB *gene of *Y. enterocolitica *biovar 1A [20]. This suggested that *blaB *gene of *Y. intermedia *and *Y. frederiksenii *was more heterogenous compared to that of *Y. enterocolitica*.

### Sequencing of *bla *genes

The *blaA *gene sequence of *Y. intermedia *showed high degree of identity with *Y. enterocolitica *biovar 1A (96.8%) [20], *Y. enterocolitica *8081 biovar 1B (90%) and *Y. enterocolitica *Y-56 biovar 4 (87.7%) [25]. The identity of *blaA *of *Y. frederiksenii *with that of the above-mentioned organisms was however found to be 77%, 79.5% and 79.8% respectively, suggesting a distinct lineage of *Y. frederiksenii*. The sequence identity of *blaA *gene of *Y. intermedia *and *Y. frederiksenii *with other members of the family *Enterobacteriaceae *namely *Klebsiella oxytoca *and *Citrobacter koseri *was comparatively very low and ranged from 48–50% for *Y. intermedia *and 62–63% for *Y. frederiksenii*. Furthermore, identity of *blaA *of *Y. intermedia *and *Y. frederiksenii *with *Burkholderia cepacia*, a non-enterobacterial species was 47.7% and 60%, respectively.

**Figure 4:**
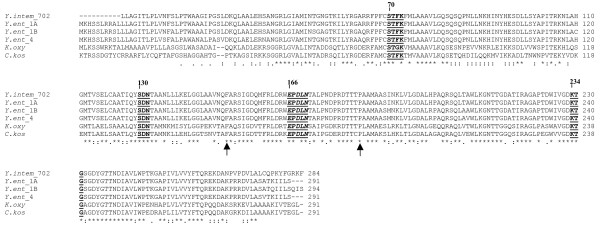
**Amino acid sequence alignment of class A β-lactamase (Bla-A) of *Y. intermedia *(strain C-702) with its nearest β-lactamase neighbors. **The partial sequence of Bla-A of *Y. frederiksenii *was not included in alignment analysis because the deduced amino acid sequence was of 121 residues only. Asterisks indicate identical amino acids. The conserved motifs (^70^SXXK^73^, ^130^SDN^132 ^and ^234^KTG^236^) typical of class A β-lactamases are underlined and in boldface. The motif ^166^EXXLN^170 ^responsible for omega-loop formation is in boldface and italics. Arrows indicate the putative omega (Ω) loop region. The GenBank accession numbers of the β-lactamases are as follows: *Y.intem*_702 (*Y. intermedia*, ABD84039, present study),*Y.ent_*1A (AAX55643, *Y. enterocolitica *biovar 1A), *Y.ent*_1B (*Y. enterocolitica *8081 biovar 1B), *Y.ent*_4 (CAA40357, *Y. enterocolitica*, Y-56 biovar 4), *K.oxy*, (AAA25084, *K. oxytoca*) and *C.kos *(CAA44485, *C. koseri*).

The deduced amino acid sequences of Bla-A of *Y. intermedia *and *Y. frederiksenii *were analyzed to check similarity in β-lactamases at protein level (Fig. 4). The SXXK tetrad, characteristic of β-lactamases possessing a serine active site, was present at position 70–73 [26]. In addition, two structural motifs characteristic of class A β-lactamases, were also found to be present in Bla-A of *Y. intermedia *and *Y. frederiksenii*: SDN at position 130–132 and KTG at position 234–236 along with the motif responsible for omega (Ω) loop formation, *i.e*., ^166^(EPDLN)^170^. The amino acid sequence alignment revealed high degree of identity (87–94%) of Bla-A of *Y. intermedia *and *Y. frederiksenii *with that of *Y. enterocolitica *biovars 1A, 1B and 4. The percent identity of Bla-A of *Y. frederiksenii *with *K. oxytoca *and *C. koseri *was found to be higher (69–71%) compared to that of *Y. intermedia *(49–53%). Overall, the data suggested that class A β-lactamase of *Y. intermedia *shared high identity with *Y. enterocolitica*, and that of *Y. frederiksenii *with other members of the family *Enterobacteriaceae *and *B. cepacia*. This seems to reiterate the distinct lineage of *Y. frederiksenii *as suggested by other investigators based on 16S rDNA and gyrase B genes sequence analyses [27].

**Figure 5:**
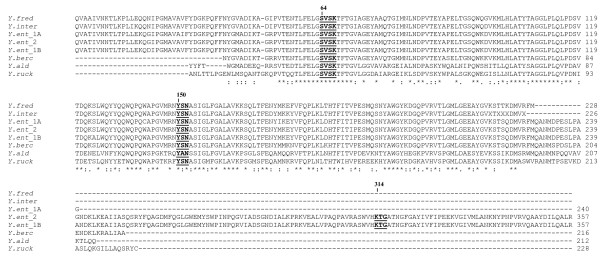
**Amino acid sequence alignment of AmpC β-lactamase (Bla-B) of *Y. intermedia *(strain C-702) and *Y. frederiksenii *(strain C-115) and the nearest class C β-lactamase neighbors. **Asterisks indicate identical amino acids. The conserved motifs (^64^SXXK^67^, ^150^YSN^152 ^and ^314^KTG^316^) typical of class C β-lactamases are underlined and in boldface. The GenBank accession numbers of the β-lactamases are as follows: *Y.fred *and *Y.inter *(ABD84040 and ABD84041, present study),*Y.ent*_1A (AAZ66331, *Y. enterocolitica *biovar 1A),*Y.ent_*2 (CAA44850, *Y. enterocolitica*, IP97 biovar 2), *Y.ent*_1B (*Y. enterocolitica *8081 biovar 1B),*Y.berc *(AAO21212, *Y. bercovieri*), *Y.ald *(AAO21211, *Y. aldovae*) and *Y.ruck *(AAM94804, *Y. ruckeri*).

The nucleotide sequences of *blaB *of *Y. intermedia *and *Y. frederiksenii *were found to have 85% identity with that of *Y. enterocolitica *biovar 1A, *Y. enterocolitica *8081 biovar 1B and *Y. enterocolitica *IP97 biovar 2, and 77% with *Y. bercovieri*. But it was only 50–55% with *Y. aldovae *and *Y. ruckeri*. The deduced amino acid sequence similarity of Bla-B of *Y. intermedia *and *Y. frederiksenii *with *Y. enterocolitica *biovars 1A, 1B and 2 ranged from 89–92%. The comparison of these sequences with that of other "*Y. enterocolitica*-like" species such as *Y. bercovieri*, *Y. aldovae *and *Y. ruckeri *showed percent identity of 80%, 53% and 49% respectively. Thus, the phylogenetic relationships as discerned by multilocus enzyme electrophoresis [28,29] were reiterated in antibiotic resistance genes, both at the nucleotide and the protein level. The deduced amino acid sequence of Bla-B also revealed presence of the three characteristic motifs viz., ^64^SXXS^67^, ^150^YAN^152 ^and ^314^KTG^316 ^(Fig. 5).

### Molecular weight determination of Bla-A and Bla-B

Molecular weight determination on SDS-PAGE showed two distinct bands of 37 kDa and 29 kDa for all strains of *Y. intermedia *and *Y. frederiksenii*. The band at 37 kDa was characteristic of Bla-A enzyme, as the molecular weight of most class A β-lactamases from other organisms namely *Y. enterocolitica *[20], *Burkholderia pseudomallei *[30] and *Citrobacter sedlakii *[31] has been reported to vary between 29–35 kDa. The identity of this band was also confirmed by specific inhibition with clavulanic acid. The band at 29 kDa corresponded to Bla-B (AmpC) as indicated by its specific inhibition with aztreonam. The molecular weight of inducible cephalosporinases or AmpC of other species namely *Y. enterocolitica *[32], *Y. aldovae*, *Y. bercovieri, Y. ruckeri *and *Y. frederiksenii *[15], and *Serratia marcescens *[33], has however been reported to be in the range of 34–40 kDa. The 29 kDa as found in the present study was similar to 29 kDa AmpC of *Vibrio fischeri *[34] and *Y. enterocolitica *biovar 1A [20]. Since most studies cited above used SDS-PAGE to determine the molecular weight, the differences cannot be attributed to methodology.

### Isoelectric focusing analysis of Bla-A and Bla-B

Isoelectric focusing of β-lactamases of fifteen strains (8 *Y. intermedia *and 7 *Y. frederiksenii*) revealed a single band in the alkaline region of the gel at pI 8.7 (Table 3) that corresponded to Bla-A as indicated by its inhibition by clavulanic acid. Tzelepi et al [35] reported that Bla-A of *Y. intermedia *focused at pIs 9.0–9.5. The Bla-A of *Y. enterocolitica *with pI 8.7 has been reported by several investigators [20,36,37].

The inducible cephalosporinase or AmpC β-lactamase focused as multiple bands in the acidic region of the gel at various pI values. The identity of these bands was confirmed both by induction with imipenem that made the bands more prominent, and also by specific inhibition with aztreonam. Two major bands in acidic region of the gel at pI 6.5 and 6.8 were observed for the clinical strains of *Y. intermedia*. The AmpC of non-clinical strains of *Y. intermedia *focused at pI ranging from 5.5 to 7.1 (Table 3). Two additional bands (pI 7.8 and 8.0) were observed in the pig throat strains. The only report on Bla-B (AmpC) of *Y. intermedia *reported pI to be between 5.5 to 6.1 [35]. The Bla-B of all the strains of *Y. frederiksenii *also appeared as multiple bands at pI 6.0, 6.8 and 8.0. The strains of *Y. enterocolitica*, for which most information is available in literature [36,37], produced Bla-B with pI 5.3–5.7 except biovar 1A strains, the Bla-B of which focused at pI 6.8 and 7.1. Consequently, the Bla-B of biovar 1A strains has been termed as "Bla-B like" [11]. The present study indicated that, like biovar 1A strains, *Y. intermedia *and *Y. frederiksenii *too produced "Bla-B like" enzyme.

### Nucleotide sequence accession numbers

The nucleotide sequence data of *blaA *and *blaB *has been submitted to NCBI GenBank under accession numbers [GenBank: DQ424965 and GenBank: DQ656113], and [GenBank: DQ424967 and GenBank: DQ424968] respectively.

## Conclusion

The two β-lactamases namely Bla-A and Bla-B were detected in all clinical and non-clinical strains of *Y. intermedia *and *Y. frederiksenii *isolated from India. Differential expression of Bla-A but not Bla-B was observed by double disc diffusion method. Both *Y. intermedia *and *Y. frederiksenii *were highly resistant to β-lactam antibiotics. PCR-RFLP revealed that *blaA *and *blaB *genes of both *Y. intermedia *and *Y. frederiksenii *were heterogeneous. Phylogenetic relationships also showed that the two species shared high degree of identity in their *bla *genes. Isoelectric focusing data revealed that both *Y. intermedia *and *Y. frederiksenii *produced Bla-A and "Bla-B like" enzymes. This is the first study in which β-lactamases (Bla-A and Bla-B) and β-lactamase genes (*blaA *and *blaB*) of *Y. intermedia *and *Y. frederiksenii *isolated from Asian region have been investigated.

## Methods

### Bacterial strains

Thirty four strains of *Y. intermedia *and fifteen of *Y. frederiksenii *isolated in India [38,39] from different sources namely diarrheic human subjects (10 strains), pig throat (31 strains) and wastewater (8 strains) were examined. The strains were authenticated by, and have been deposited with the *Yersinia *National Reference Laboratory and WHO Collaborating Centre at Institut Pasteur (Paris), France. The details of the strains are given in Table 4.

### Antibiotics and chemicals

Mueller-Hinton agar (MHA), tryptone glucose yeast extract (TGYE) agar, tryptone soya broth (TSB) and antibiotic discs, namely ticarcillin 75 μg and imipenem 10 μg were obtained from HiMedia, Mumbai (India). Co-amoxyclav 3 μg (containing 2 μg amoxicillin and 1 μg clavulanate) and cefotaxime 5 μg discs, and nitrocefin were purchased from Oxoid (Basingstoke, UK). Amoxicillin and ceftazidime were purchased from HiMedia. Cefotaxime, co-amoxiclav (augmentin) and cefepime (megapime) were from Nicholas Piramal India Ltd., GlaxoSmithKline Pharmaceuticals Ltd and Alkem Laboratories Ltd, Mumbai (India) respectively.

### Phenotypic detection of Bla-A and Bla-B enzymes

Detection of the enzymes Bla-A and Bla-B was carried out by double disc diffusion tests as described previously by Pham et al [9] and Pham and Bell [40] respectively. Briefly, culture grown overnight on TGYE agar was used to prepare a suspension of the test organism containing 10^7 ^CFU/ml (A_600 _= 0.1). MHA plates were inoculated by flooding these with 2.5 ml of this suspension. The excess was removed and plates were allowed to dry. For detection of Bla-A, ticarcillin 75 μg and co-amoxiclav 3 μg discs were placed on the plate, with adjacent edges 22 mm apart. The plates were incubated at 28°C for 20 hours. After incubation, the radii and diameter of the zone of inhibition around ticarcillin disc were recorded. The plates were also observed for presence of synergy (an additional zone of inhibition) between ticarcillin and co-amoxiclav discs. For detection of enzyme Bla-B, cefotaxime 5 μg and imipenem 10 μg discs were placed similarly on MHA plate and incubated. The characteristic flattening of the zone of inhibition around cefotaxime disc adjacent to the imipenem disc was interpreted as presence of Bla-B.

### Minimum Inhibitory Concentrations (MIC)

The MICs of five selected antibiotics namely amoxicillin, co-amoxiclav, cefotaxime, ceftazidime and cefepime were determined in Mueller-Hinton broth by microbroth dilution technique using the methodology described by the Working Party of the British Society for Antibacterial Chemotherapy [41].

### PCR amplification of *blaA *and *blaB *genes

Total genomic DNA from each strain was extracted by DNA extraction kit (Qiagen, Germany) as per the manufacturer's instructions. PCR amplification of *bla*A gene from all strains of *Y. intermedia *and *Y. frederiksenii *was performed using the primers, A7-f (5' TATGCCCCGATCACGCGTAAAAATCT 3') and A8-r (5' CAAAGTACCGCAATATCATTGGTGGT 3'). These were designed by aligning sequences of *blaA *genes of *Y. enterocolitica *biovar 1A [20], *Y. enterocolitica *8081 biovar 1B [42] and *Y. enterocolitica *Y-56 biovar 4 [25] and the expected amplicon size was 450 bp. The *blaB *gene was amplified using published primers blaB5 (5'CCCACTTTATACCTTGGCACAAA 3') and blaB3 (5'GAACATATCTCCTGCCTGCAAAT 3'), which amplified a fragment of 827 bp [10]. Cycling conditions for *blaA *included: initial denaturation at 95°C for 5 min followed by 25 cycles each at 54°C for 30 sec, 72°C for 90 sec and 95°C for 30 sec, and a final extension at 72°C for 10 min. For the *blaB*, cycling conditions for PCR were as described by Stock et al. [10]. The PCR products were electrophoresed on 1% agarose gel in 1 × TAE (40 mM Tris acetate, 1 mM EDTA, pH 8.0) buffer at a constant voltage of 80 V/cm.

### PCR-Restriction Fragment Length Polymorphisms of *bla *genes

PCR-RFLP of *blaA *was carried out with NciI and HaeIII, and that of *blaB *with HaeIII and RsaI (New England Biolabs Inc., Schwalbach, Germany) as per manufacturer's instructions. The digestion mixture was incubated at 37°C for 8 hours and resolved on 2% agarose gel in 1 × TBE (Tris-Borate-EDTA). The gels were stained with ethidium bromide (0.5 μg/ml) and photographed under UV illumination. Similarity amongst *blaA *and *blaB *was estimated by cluster analysis of the restriction profiles of each gene using Jaccard's similarity coefficient and a UPGMA (Unweighted Pair Group Method with Arithmetic mean) dendrogram was constructed separately for each gene using Diversity Database software (Bio-Rad, USA).

### Sequencing of *blaA *and *blaB *genes

The complete CDS of *blaA *gene (896 bp) of *Y. intermedia *was amplified with primers A9-f and A10-r [20]. However, CDS of *blaA *of *Y. frederiksenii *could not be amplified using these primers. Thus, partial sequence of *blaA *(450 bp) of *Y. frederiksenii *was amplified using primers A7-f and A8-r. The amplicons of *blaA *and *blaB *genes of representative strains, each of *Y. intermedia *and *Y. frederiksenii*, were purified using QIA Quick Gel Extraction Kit (Qiagen, Germany) and sequenced by Big Dye Terminator Cycle Sequencing Ready Reaction kit using ABI 310 Genetic Analyzer (Applied Biosystems, Germany). The sequences obtained were analyzed for homology with *bla *genes in the existing GenBank database using Blastn [43]. The deduced amino acid sequences of the proteins were aligned with class A (for Bla-A) and class C (for Bla-B) β-lactamases of other members of the family *Enterobacteriaceae *using ClustalW [44].

### Molecular weight determinations of Bla-A and Bla-B enzymes

The molecular weights of the two β-lactamases were determined on SDS-PAGE. Crude cell lysate containing enzymes was prepared by sonication of washed cell pellet as described earlier [8]. For induction of enzyme Bla-B, imipenem (final concentration 0.5 mg/l) was added during the log phase of cell culture. The protein concentration was estimated by Lowry's method [45]. Sodium dodecyl sulfate-polyacrylamide gel electrophoresis (SDS-PAGE) was performed as described by Laemmli [46]. Briefly, 50 μg of protein was loaded on 12% resolving and 5% stacking SDS-polyacrylamide gel (Mini Protean III, Bio-Rad, USA) along with the medium range molecular weight protein marker (Bangalore Genei, India). The electrophoresis was carried out at 80 V for 2 hours. For renaturation, the gels were washed twice, for 45 min each, in renaturation buffer (100 mM Tris HCl, pH 7.0 and 0.1% Triton-X-100) under mild shaking. β-lactamase bands were visualized by overlaying the gels with 0.5 mg/ml nitrocefin for 2 min. The identity of the bands was further confirmed by specific inhibition with 40 μM clavulanic acid for Bla-A or 20 μM aztreonam for Bla-B.

### Isoelectric focusing (IEF) of Bla-A and Bla-B enzymes

IEF of the crude enzyme extract (3 μg of protein) was performed in 6% polyacrylamide gel containing 2% ampholyte of pH 3 to 10 (Biolyte Ampholyte, Bio-Rad, USA) using Mini IEF cell (Bio-Rad, USA) according to the protocol specified by the manufacturer. A broad range IEF standard with pI ranging from 4.45–9.6 (Bio-Rad, USA) was used as pI marker. The β-lactamase bands were visualized by overlaying the IEF gel with nitrocefin (0.5 mg/ml). The identity of the bands was further confirmed by specific inhibition with clavulanic acid and aztreonam as described above.

## Authors' contributions

SM (first author) carried out the major part of the work namely PCR amplification of the genes, PCR-RFLP, MIC, IEF, analysis and interpretation of data, and drafted the manuscript. SM (second author) and SS carried out disc diffusion tests for detection of β-lactamases and participated in molecular weight determination and IEF. JSV conceived the study, coordinated and supervised the work and helped in drafting the final manuscript. All authors have read and approved the manuscript.

## Figures and Tables

**Table 1:** Detection of β-lactamases Bla-A and Bla-B in *Y. intermedia *and *Y. frederiksenii *isolated from India

Species	Source (n)	Detection of Bla-A		Detection of Bla-B
		
		Number positive^a ^(%)	Synergy^b ^(%)	Number positive^c ^(%)
*Y. intermedia*	Clinical (4)	4 (100)	-	4 (100)
	Wastewater (8)	8 (100)	4 (50)	8 (100)
	Pig throat (22)	22 (100)	5 (22.7)	22 (100)
*Y. frederiksenii*	Clinical (6)	6 (100)	-	6 (100)
	Pig throat (9)	9 (100)	1 (11.1)	9 (100)

n number of strains

^a^number of strains with annular radii between 2–8 mm (diameter 10–22 mm) of the zone of inhibition around ticarcillin disc

^b^Synergy – additional zone of inhibition between ticarcillin and co-amoxiclav discs

^c^number of strains that showed characteristic flattening of the zone of inhibition around cefotaxime disc.

**Table 2:** Susceptibility of *Y. intermedia *and *Y. frederiksenii *to selected antibiotics

**Source (n)**	**MIC_50_(mg/L)**					**MIC_90_(mg/L)**				
	
	**AMX**	**AMC**	**CTX**	**CAZ**	**FEP**	**AMX**	**AMC**	**CTX**	**CAZ**	**FEP**
Clinical (10)	512	128	64	512	512	1024	256	128	1024	1024
Non-clinical (39)^a^	1024	256	32	512	256	2048	512	128	1024	512

n Number of strains; AMX, Amoxicillin; AMC, Co-amoxiclav; CTX, Cefotaxime; CAZ, Ceftazidime; FEP, Cefepime

^a^Watsewater (8 strains) and Pig throat (31 strains)

**Table 3:** Isoelectric points (pI) of β-lactamases (Bla-A and Bla-B) of *Y. intermedia *and *Y. frederiksenii*

Strains	n	pI	
		
		Bla-A	Bla-B
*Y. intermedia*			
Clinical	4	8.7	6.5, 6.8, 7.5
Non-clinical^a^	4	8.7	6.0, 6.8, 7.1, 7.8, 8.0
			5.5, 6.0
			6.8, 7.0

*Y. frederiksenii*			
Clinical	6	8.7	6.0, 6.8, 8.0
Non-clinical^b^	1	8.7	6.0, 6.8, 8.0

n: number of strains

^a^Wastewater (2 strains) and pig throat (2 strains)

^b^Isolates from pig throat

**Table 4:** Biotypes, serotypes and sources of strains of *Y. intermedia *and *Y. frederiksenii *used in this study

**S. No.**	**Strain No.^a^**	**Species**	**Serotype**	**Biotype**	**Source**	**Ref. Lab no.**
1	K/Y/30	*Y. intermedia*	O:40	4	Wastewater	IP26142
2	K/Y/31	*Y. intermedia*	O:40	4	Wastewater	IP 26143
3	T/Y/33	*Y. intermedia*	NAG	1	Wastewater	IP 26259
4	O/Y/43	*Y. intermedia*	O:40	4	Wastewater	IP 26146
5	Ko/Y/57	*Y. intermedia*	NAG	2	Wastewater	IP 26306
6	R/Y/59	*Y. intermedia*	O:40	4	Wastewater	IP 26309
7	O/Y/60	*Y. intermedia*	O:40	4	Wastewater	IP 26 308
8	T/Y/65	*Y. intermedia*	O:40	4	Wastewater	IP 26318
9	C-594	*Y. intermedia*	NAG	2	Clinical	IP 27477
10	C-702	*Y. intermedia*	7,8-8	1	Clinical	IP 72478
11	C-714	*Y. intermedia*	NAG	1	Clinical	IP 27479
12	C-752	*Y. intermedia*	NAG	1	Clinical	IP 27480
13	P-60	*Y. intermedia*	-	-	Pig throat	-
14	P-114	*Y. intermedia*	-	-	Pig throat	-
15	P-122	*Y. intermedia*	-	-	Pig throat	-
16	P-144	*Y. intermedia*	-	-	Pig throat	-
17	P-185	*Y. intermedia*	-	-	Pig throat	-
18	P-225	*Y. intermedia*	-	-	Pig throat	-
19	P-226	*Y. intermedia*	-	-	Pig throat	-
20	P-262	*Y. intermedia*	-	-	Pig throat	-
21	P-337	*Y. intermedia*	-	-	Pig throat	-
22	P-360	*Y. intermedia*	-	-	Pig throat	-
23	P-364	*Y. intermedia*	-	-	Pig throat	-
24	P-366	*Y. intermedia*	-	-	Pig throat	-
25	P-368	*Y. intermedia*	-	-	Pig throat	-
26	P-369	*Y. intermedia*	-	-	Pig throat	-
27	P-387	*Y. intermedia*	-	-	Pig throat	-
28	P-396	*Y. intermedia*	-	-	Pig throat	-
29	P-402	*Y. intermedia*	-	-	Pig throat	-
30	P-430	*Y. intermedia*	-	-	Pig throat	-
31	P-459	*Y. intermedia*	-	-	Pig throat	-
32	P-460	*Y. intermedia*	-	-	Pig throat	-
33	P-468	*Y. intermedia*	-	-	Pig throat	-
34	P-474	*Y. intermedia*	-	-	Pig throat	-
35	C-115	*Y. frederiksenii*	NAG	-	Clinical	IP27388
36	C-203	*Y. frederiksenii*	35	-	Clinical	IP27389
37	C-249	*Y. frederiksenii*	35	-	Clinical	IP27390
38	C-503	*Y. frederiksenii*	35	-	Clinical	IP27399
39	C-567	*Y. frederiksenii*	35	-	Clinical	IP27401
40	C-580	*Y. frederiksenii*	35	-	Clinical	IP27402
41	P-180	*Y. frederiksenii*	-	-	Pig throat	-
42	P-236	*Y. frederiksenii*	-	-	Pig throat	-
43	P-238	*Y. frederiksenii*	-	-	Pig throat	-
44	P-240	*Y. frederiksenii*	-	-	Pig throat	-
45	P-257	*Y. frederiksenii*	-	-	Pig throat	-
46	P-259	*Y. frederiksenii*	-	-	Pig throat	-
47	P-263	*Y. frederiksenii*	-	-	Pig throat	-
48	P-270	*Y. frederiksenii*	-	-	Pig throat	-
49	P-273	*Y. frederiksenii*	-	-	Pig throat	-

^a ^All strains were isolated from New Delhi, India

NAG: Non-agglutinable

IP: *Yersinia *National Reference Laboratory and WHO Collaborating Centre, Institut Pasteur, Paris (France)
